# The Influence of the Trigeminus Nerve on the Organs of Hearing

**Published:** 1883-02

**Authors:** 


### THE INFLUENCE OF THE TRIGEMINUS NERVE ON THE ORGANS OF HEARING.

Dr. Kirchner, in his experiments on the fifth pair of nerves, attempts to show that diseases of the ears may be due to injuries of the branches of the trigeminus. After referring to the experiments of Gelle, Hagen and Berthold on the vaso-motor disturbances of the tympanic cavity, he turns his attention to the third branch of the trigeminus, because filaments of this nerve are distributed to the pharynx and also inervate the tensor bel palati, probably the most important of the tubal muscles.

Dr. Kirchner demonstrates that diseases of the pharynx have an etiological relation to diseases or disturbances of the drum-head on account of the peculiar nerve distribution. With the assistance of Dr. Ashenbrandt, the author made a number of experiments on animals, laying bare the third branch of the fifth nerve in such a way as to expose the tympanic cavity at the same time, so that direct observations could be made. The condition of the mucous membrane with regard to its vascular supply and the secretion of mucous could be accurately studied while the nerve itself was being cut, later on, when stimulated by electricity. When a weak current was used for about ten seconds the delicate network of vessels in the tympanic mucous membrane could be seen to enlarge and remain distinctly visible for a few seconds, even after electrization had been discontinued. After the application of a strong current for a longer time there was a distinctive congestion of the capillary network, the color of the mucous membrane increased to a livid red, which disappeared only after a considerable length of time. Besides these changes, Dr. Kirchner states, when a very strong current was used, a thin, watery exudation made its appearance on the mucous membrane of the tympanic cavity, and when the irritation ceased the exudation disappeared. When the fifth nerve was injured by pulling out its root near the base of the skull, the tympanic mucous membrane became swollen and secreted a muco-purulent matter in the course of a few weeks after the injury. These experiences may be explained in the following manner: irritation of the third branch of the fifth nerve might excite the nerve-fibres controlling secretion, by a reflex action, or the strong injection of the capillary system that occurs at the same time causes an increase of secretion, or the otic ganglion may be affected through the nerve filaments running from the tympanic cavity to the ganglion, and in this manner influence the secretory as well as the circulatory condition. Aside from the scientific value of these experiments, they may be of practical benefit to the physician, enabling him to treat more successfully a disease of the ear when arising after an injury to the trigeminus. —*Lancet and Clinic.*

